# Breast Cancer Predisposition Genes and Synthetic Lethality

**DOI:** 10.3390/ijms22115614

**Published:** 2021-05-25

**Authors:** Hannah E. Neiger, Emily L. Siegler, Yihui Shi

**Affiliations:** 1 College of Graduate Studies, California Northstate University, Elk Grove, CA 95757, USA; Hannah.Neiger6774@cnsu.edu; 2 College of Medicine, California Northstate University, Elk Grove, CA 95757, USA; Emily.Siegler6569@cnsu.edu

**Keywords:** *BRCA1*/*BRCA2*, CPGs, PARPi, hereditary breast cancer, synthetic lethality, DNA repair

## Abstract

*BRCA1* and *BRCA2* are tumor suppressor genes with pivotal roles in the development of breast and ovarian cancers. These genes are essential for DNA double-strand break repair via homologous recombination (HR), which is a virtually error-free DNA repair mechanism. Following *BRCA1* or *BRCA2* mutations, HR is compromised, forcing cells to adopt alternative error-prone repair pathways that often result in tumorigenesis. Synthetic lethality refers to cell death caused by simultaneous perturbations of two genes while change of any one of them alone is nonlethal. Therefore, synthetic lethality can be instrumental in identifying new therapeutic targets for *BRCA1/2* mutations. PARP is an established synthetic lethal partner of the *BRCA* genes. Its role is imperative in the single-strand break DNA repair system. Recently, Olaparib (a PARP inhibitor) was approved for treatment of *BRCA1/2* breast and ovarian cancer as the first successful synthetic lethality-based therapy, showing considerable success in the development of effective targeted cancer therapeutics. Nevertheless, the possibility of drug resistance to targeted cancer therapy based on synthetic lethality necessitates the development of additional therapeutic options. This literature review addresses cancer predisposition genes, including *BRCA1*, *BRCA2*, and *PALB2*, synthetic lethality in the context of DNA repair machinery, as well as available treatment options.

## 1. Introduction

Breast cancer is the most commonly diagnosed cancer type in women and ranks highest in mortality rate, constituting 11.6% of global cancer deaths alone each year [1]. Risk factors for developing breast cancer include increased exposure to estrogen conferred by early menarche, late menopause, and late parity; lifestyle factors such as high alcohol consumption and obesity; and genetic predisposition [2]. Approximately 5% to 10% of breast cancer is hereditary. Genes in which germline mutations confer increased risk of cancer are called cancer predisposition genes (CPGs). Of breast cancer cases, Breast Cancer Gene 1 (*BRCA1*) and Breast Cancer Gene 2 (*BRCA2*) mutations are the most commonly encountered CPGs [3]. Approximately 50% of all hereditary breast cancer cases are due to *BRCA1/2* CPG mutations and are associated with early-onset breast cancer [4].

Although mutations in *BRCA1* and *BRCA2* are two of the most widely known genetic determinants of breast and ovarian cancer, they are among many in a series of CPGs implicated in the formation of hereditary breast cancer (Table 1). Partner and Localizer of *BRCA2* (*PALB2*) is a caretaker gene implicated in both *BRCA1/2* breast cancer and Fanconi’s Anemia (FA) in the event of monoallelic and biallelic loss-of-function mutations, respectively [5]. Additional breast CPGs implicated in moderate to severe breast cancer risk include *BRCA1* Associated Ring Domain 1 (*BARD1*) [6], Tumor Protein P53 (*TP53*) [7], *BRCA1* Interacting Protein 1 (*BRIP1*) [7], and *RAD51* Paralog C (*RAD51C*) [8]. Interestingly, several of the described breast CPGs that confer significant risk are involved in DNA repair pathways and cell cycle checkpoint mechanisms. 

Considerable advancements in molecular biology and therapeutics have been made in the 25 years following the discovery of tumor suppressor genes *BRCA1* and *BRCA2* and their roles in the development of breast and ovarian cancer. Their involvement in the development of pancreatic, prostate, gastrointestinal, and skin cancers has also been demonstrated in both men and women [16]. The global association of *BRCA1/2* mutations with diverse forms of cancer is likely due to the crucial function of both *BRCA1* and *BRCA2* in DNA repair systems, which are cornerstone to physiological functionality and maintenance of cells throughout the body. Both *BRCA1* and *BRCA2* are essential genes in DNA double-strand break (DSB) repair via the homologous recombination (HR) pathway, which is a virtually error-free DNA repair mechanism [17,18]. HR is directly compromised in the event of deleterious germline mutations on chromosome 17 (*BRCA1*) or chromosome 13 (*BRCA2*), forcing cells to resort to alternative methods of DSB repair through error-prone pathways, allowing accumulation of damage and ultimately expediting tumorigenesis [6].

A challenge faced by research in the pursuit of effective therapies is the inability to target mutated *BRCA1/2* directly. *BRCA1/2* are considered “undruggable” target genes because mutations in *BRCA1/2* lead to loss of BRCA1/2 function [19]. This problem is not one commonly faced by other types of cancers, especially those driven by constitutive action or gain-of-function mutations in oncogenes, e.g., HER2 breast cancers. Given this context of *BRCA1/2*-mutated cancers, an obstacle is posed in creating an effective therapeutic strategy: how do we target the un-targetable? The answer points to synthetic lethality. Synthetic lethality is a system defined by the maintenance of cell viability when one of two genes within two interdependent survival pathways is silenced, while silencing both genes would lead to cell death [20]. The two genes in this system are denoted “synthetic lethal partners.” This system is especially useful for targeting and killing malignant cells with deleterious mutations, for which *BRCA1* and *BRCA2* are excellent candidates because of their roles in DSB repair. A previously elucidated synthetic lethal partner of the *BRCA* genes is Poly ADP ribose polymerase (PARP), whose role is imperative in the single-strand break (SSB) DNA repair system, specifically base excision repair [21]. The additive effect of impaired DSB repair and a stalled SSB repair system yields cancer cells with no available survival pathway, effectively targeting oncogenic cells. 

In 2018, a PARP inhibitor, Olaparib, received full FDA approval for the treatment of *BRCA1/2*-mutated breast and ovarian cancer, becoming the first therapy to utilize the principle of synthetic lethality, marking a considerable step forward in the pursuit of effective cancer therapeutics [22]. Research currently focuses on further elucidating other synthetic lethal partners of *BRCA1* and *BRCA2* for drug development, in addition to developing more effective inhibitors, immunomodulatory drugs, and other targeted treatments. This literature review addresses cancer predisposition genes, including *BRCA1*, *BRCA2*, and *PALB2*; synthetic lethality in the context of DNA repair machinery; and historical and current treatment options for breast and ovarian malignancies with these mutations.

## 2. Breast CPGs Affecting HR

*BRCA1*, *BRCA2*, *PALB2*, and *BARD1* are critical to proper function of the HR repair pathway and cell cycle checkpoints. These are all considered high-risk breast cancer susceptibility genes, for which mutations or abnormal characteristics increase incidence of oncologic activity. *TP53*, *BRIP1*, and *RAD51C* are also involved in cell cycle checkpoints and HR, and are associated with moderate breast cancer risk due to their relatively accessory roles in this repair mechanism [7,8]. Further analysis of HR and other DSB and SSB DNA repair pathways will follow in this discussion about breast CPGs.

**Figure 1:**
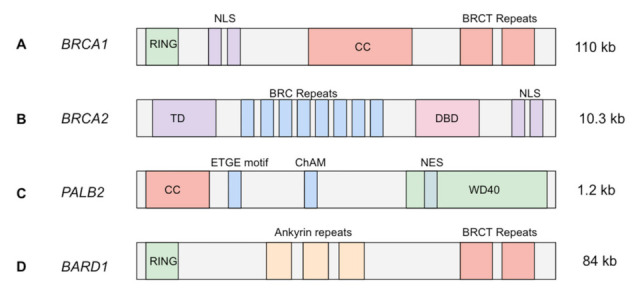
Transcript structure of high-risk CPGs. (**A**) A 110 kb full-length transcript of *BRCA1*, including N-terminus RING domain, nuclear localization sequence (NLS), coiled-coil domain (CC), and two BRCA1 C-terminus (BRCT) repeats [28,29,30]; (**B**) a 10.3 kb transcript of *BRCA2*, including N-terminus transactivation domain (TD), eight BRC repeats, DNA-binding domain (DBD), and C-terminus NLS [28,30,31,32]; (**C**) a full-length 1.2 kb *PALB2* transcript, with coiled-coil domain (similar to that in *BRCA1*), ETGE motif, chromatin association motif (ChAM), and the WD40 repeat region, which includes a nuclear export signal (NES) [31,32,33]; (**D**) a *BARD1* 84 kb transcript, including an N-terminus RING domain (similar to that in *BRCA1*), three ankyrin repeats, and two BRCT repeats on the C-terminus (similar to that in *BRCA1*) [34,35,36,37].

BRCA1 plays a vital role in several critical cell processes including growth, division, transcriptional regulation, DNA repair, and apoptosis [23]. In HR, BRCA1 is the backbone of the HR complex. BRCA1 itself consists of four main functional domains: Really Interesting New Gene (RING), coiled-coil domain, and the two BRCA1 C-terminus (BRCT) domains (Figure 1A). The RING domain of BRCA1 interacts with a similar domain on the N-terminus of BARD1, an E3 ubiquitin ligase. This interaction ultimately removes P53-Binding Protein-1 (53BP1), allowing HR to proceed over the less precise NHEJ repair pathway [24]. The coiled-coil domain binds a complementary domain in PALB2, assisting in the process of complex loading to the site of DSB; BRCT motifs interact with several other proteins involved in HR and function as the site of BRCA1 complexation [25]. In the event that BRCA1 loses function, such as in cells harboring deleterious germline mutations, HR cannot proceed, shunting DNA repair to the NHEJ pathway as the primary repair mechanism of DSBs. Through its BRCT domain, BRCA1 interacts with a number of additional proteins, which accounts for the its involvement in the aforementioned cell processes (growth, division, transcriptional regulation, and apoptosis) [26]. Most tumorigenic germline mutations in *BRCA1* occur in either the RING domain (interacting with BARD1) or the BRCT domains (which interact with several proteins), ultimately impeding the HR pathway [27].

*BRCA2* is located on chromosome 13 and consists of one primary functional domain that includes 8 repeated motifs, denoted BRCs (Figure 1B). Each of the eight BRC motifs directly bind RAD51 and chaperone the protein to the site of damage; RAD51 subsequently activates single-strand DNA coating and invasion into the sister chromatid during HR. RAD51 catalyzes the primary step in HR; therefore, its function is critical to this repair mechanism [38]. BRCA2 additionally associates with paralogs of RAD51 at the site of DSBs; however, the explicit interactions between BRCA2 and RAD51 have not yet been well elucidated [39]. Though BRCA2 is important in successful execution of HR, BRCA1 has demonstrated the ability to recruit surrogate proteins to the DSB site (e.g., Rap80/Abraxas/BRCA1 complex) to compensate for decreased or loss of function in *BRCA2*; this supports findings that some, albeit reduced, HR still occurs in the event of deleterious germline *BRCA2* mutations, combating the accumulation of DNA damage [27]. Like BRCA1, BRCA2 has been shown to interact with cell cycle checkpoints and other critical cell processes independent of its role in HR, for instance DNA replication. This highlights *BRCA2* as an important chromosomal caretaker gene [40].

*PALB2* (also known as *FANCN*) is located on chromosome 16 and is involved in the mechanisms of HR in addition to the FA intrastrand crosslinking (ICL) pathway. In HR, the PALB2 protein associates with BRCA1 at the site of DSB in the event that DNA damage is detected. PALB2 then mediates recruitment and loading of both BRCA2 and RAD51 to the BRCA1 complex [6,41]. Defective or silenced *PALB2* in the context of HR leads to inappropriate assembly of the BRCA1/BRCA2/RAD51 complex that immediately precedes homologous strand invasion into the sister chromatid, specifically preventing the association between BRCA1 and BRCA2 [42,43]. In the absence of DNA damage, approximately half of available PALB2 and BRCA2 are still complexed in the nucleus, suggesting PALB2 mediation of the nuclear concentration of BRCA2, as well as mediation of BRCA2’s identified chromosomal caretaker functions [44]. The FA ICL pathway is predominantly used to ameliorate crosslinking events occurring during DNA replication. Here, PALB2 along with other HR proteins are recruited to initiate an HR-like repair after crosslinked DNA has been dissociated and fragmented DNA has been removed [42]. In addition to PALB2, the FA ICL and HR repair pathways share several functional proteins, most notably ATR, BRCA1, and RAD51 [45].

*BARD1* is found on chromosome 2 and is expressed in most tissues. BARD1 is a ubiquitin ligase and contains a RING domain on the N-terminus, three repeated ankyrin motifs, and two BRCT domains located on the C-terminus (Figure 1C) [46]. In the presence of DNA damage or hypoxia, *BARD1* expression is upregulated; this process occurs via cell cycle-dependent phosphorylation [47]. Mutated *BARD1* expresses a truncated protein in breast, ovarian, and uterine tissues [47]. BARD1 is the heterodimeric counterpart of BRCA1, which explains the matching nature of their N- and C-termini: the RING domain and the two BRCT domains, respectively [48]. The heterodimeric BRCA1/BARD1 complex is critical to the initiation of HR. Demonstrating its ubiquitin ligase properties, BRCA1/BARD1 promotes the transfer of ubiquitin, which results in displacement of 53BP1 [49]. Repositioning 53BP1 allows ATM to detect the DSB site and begin resection, followed by BRCA1 complexation and activation of the HR pathway [50]. Because of the ubiquitin ligase activity of BARD1, it plays a determining role in the cell’s definitive fate and whether the cell ultimately employs HR or NHEJ when responding to DSBs. The consequence of a debilitated BARD1 protein is failure to remove 53BP1, shifting DSB repair to NHEJ from failure to allow ATM HR initiation.

BRIP1, also known as BRCA1-associated C-terminal helicase (BACH1), is a DNA helicase that associates directly with the BRCT repeat domain of BRCA1 in HR [51,52]. *BRIP1* is found on chromosome 17 and contains seven helicase family-specific domains (homology boxes) and a C-terminal BRCA1-binding domain (Figure 1D) [53]. Because BRIP1 associates with the BRCT domains of BRCA1, this interaction is one of many protein interactions affected in the event of *BRCA1* germline mutations. Further, if *BRIP1* itself harbors a mutation in the Lys-52 ATP-binding pocket coding region, a missense mutation will result in lysine-52 exchanged for arginine. Such a mutation was observed to significantly decrease DSB repair activity, specifically HR [54]. Interestingly, successful BRCA1/BRIP1 interaction is contingent on the phosphorylation status of BRIP1, which in turn is dependent on the G2/M checkpoint, a DNA damage checkpoint induced by ATM [52]. Deleterious mutations to *BRIP1* will likely negatively affect the ability of the HR system to repair DSBs due to BRIP’s helicase activity in association with BRCA1; however, with regard to tumorigenesis, this perturbation is less threatening relative to those of the high-risk breast CPGs.

## 3. DNA Repair Systems

DNA repair systems utilize a variety of mechanisms and are crucial to the stability of a cell’s chromosomes via maintenance of sequence and structure. These repair mechanisms are tightly regulated and carefully orchestrated to keep pace with the natural rate of errors that occur during DNA replication; additionally, they must respond to exogenous stressors that may alter the encoding of genes. Well-studied direct and indirect candidate cancer-causing agents that introduce a need for intact and precise DNA repair include heterocyclic aromatic amines, polycyclic aromatic hydrocarbons, ultraviolet agents, nitrosamines, ionizing radiation, and asbestos [55]. A compounding effect resulting from innate impairment of DNA repair due to CPG mutation, followed by exposure to high-risk carcinogenic agents such as those listed above, will predispose patients significantly for developing cancer due to failed DNA damage repair, which is increasingly warranted with recurrent exposure to cancer-causing agents. DNA damage can be categorized into two overarching classes of breakage: DSBs and SSBs.

DSBs occur as a result of a variety of genomic insults including environmental stressors (e.g., ionizing radiation) or internally-induced lesions related to replication [56]. In all cases, two primary pathways comprise the DSB damage response in cells, HR and NHEJ. Depending on the nature of the damage, the cell initiates the more appropriate or more available pathway. Improper execution of either DSB repair mechanism can lead to pathologies that manifest in a variety of ways, ranging from developmental abnormalities to serious carcinogenesis [57]. Proper implementation and signaling of DSB repair pathways are key for maintaining genomic stability and preventing pathogenesis.

**Figure 2:**
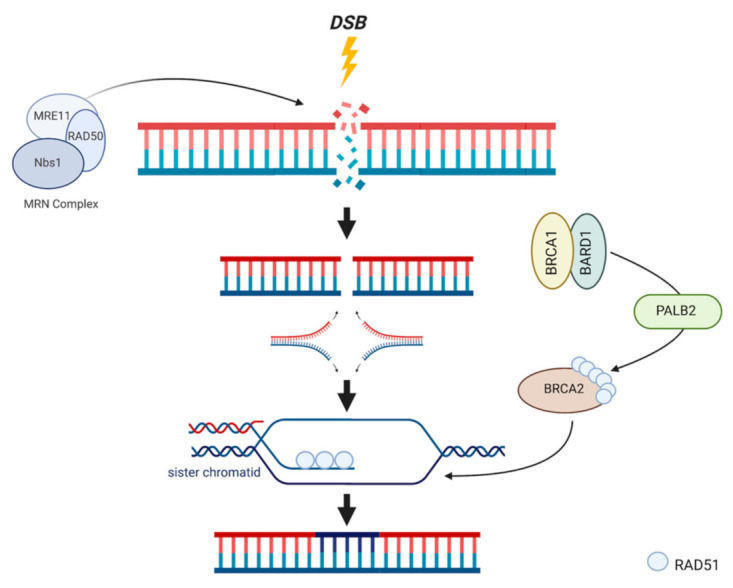
HR mechanism of action. DSBs are recognized by the MRN complex, initiating BRCA1 activation. BRCA1 and BARD1 bind via their identical RING domains to form a heterodimer which localizes to the site of damage following excision of damaged bases by an exonuclease. PALB2 is recruited to form the BRCA1/BARD1/PALB2 complex, which is responsible for recruiting BRCA2/RAD51. After DNA unwinding and dissociation, RAD51 is unloaded onto the damaged strand, and leads this strand to its available identical sister chromatid. Invasion into the sister chromatid facilitates template polymerization and subsequent ligation.

HR utilizes the identical genetic information found on the homologous sister chromatid as a temporary template to replace nucleotides of damaged DNA in the event of replication fork stalling during S-phase DNA replication [58]. A series of recruited molecules orchestrate invasion of the sister chromatid by separating the two strands, polymerizing a copy of one template strand, excising the damaged region of DNA from the original chromatid, and inserting the copied genetic material (Figure 2) [59]. Because this pathway relies entirely on the availability of the homologous sister chromatid, this mechanism of repair is cell cycle dependent, namely utilized during the S and G2 phases [60]. Commitment to the HR pathway is also dependent on the recruitment of several proteins including BRCA1, BRCA2 and the downstream products of other breast CPGs previously discussed. Evidence of the reliance of HR on BRCA1 is demonstrated by specific localization of BRCA1 at the site of DSBs [61]. Further, BRCA1 is implicated in the recruitment and complexation of several proteins involved in resection of damaged DNA in DSBs, in addition to other steps of HR. The BRCA1/BARD1 heterodimer first repositions 53BP1 from the ends of the DSB region, allowing the initiation of end resection and subsequently HR [62]. The complex then physically associates with PALB2 to recruit the BRCA2/RAD51 complex. At the breakage site, RAD51 guides the separated single strand to invade the intact sister chromatid and begin template polymerization [31,43]. In addition, BRCA1 further promotes HR by preventing single-strand ligation, is involved in replication fork re-stabilization after collapse, and amplifies the enzymatic recombinase activity of RAD51 [63,64]. The HR repair pathway is a powerful mechanism for cell survival and maintenance of genomic integrity because it effectively repairs DSBs without overlooking or accepting errors. Loss or impaired function of these proteins severely debilitates HR and forces the cell to employ a different major DSB repair mechanism, NHEJ. 

**Figure 3:**
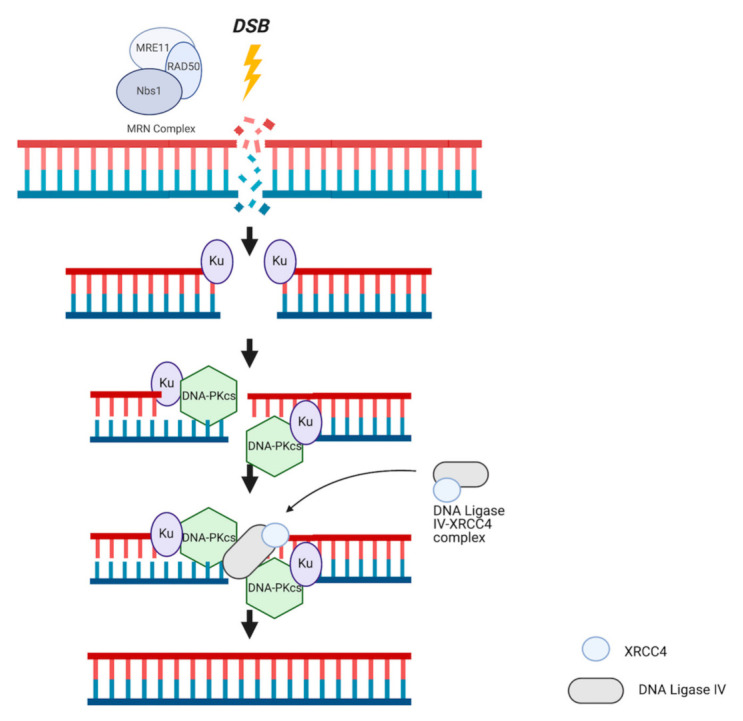
NHEJ mechanism of action. Recognition of DSBs by the MRN complex activates Ku heterodimer-mediated demarcation of the breakage site. Ku recruits DNA-PKcs to form DNA-PK complex, a number of bases are excised around the site of damage, and DNA overhangs are created. The DNA ligase-XRCC4 complex is recruited to facilitate ligation of overhangs with the assistance of additional molecules (not shown).

Alternatively, the NHEJ pathway is less apt in preserving genomic accuracy and is therefore considerably more error prone; however, it is more flexible in terms of cell cycle dependency and kinetic favorability [65]. Because this pathway does not utilize a homologous chromosome and therefore does not require sister chromatid availability, NHEJ can be implemented during any phase of the cell cycle [66]. NHEJ is characterized by three major steps: the creation of nucleotide overhangs at the site of DSB, polymerization of a small number of complementary bases following the ends of the overhangs, and subsequent ligation of free ends to complete the repair (Figure 3) [65]. Specifically, this pathway relies on two protein complexes: DNA-PK and DNA ligase IV-XRCC4. Composed of DNA-dependent protein kinase cs (DNA-PKcs) and a Ku heterodimer, this complex is responsible for recognizing the break and employing kinase activity required for self-activate and activation of additional proteins [67,68,69]. The following step includes the assembly of the DNA ligase IV-XRCC4 complex, which occurs in response to recruitment by DNA-PK. This second complex is responsible for rejoining the ends of damaged DNA; XRCC4 activates the enzymatic activity of the ligase component, while promoting its stabilization at the site of DSBs [70,71]. Though NHEJ is a conserved pathway of repair present in all eukaryotes, a property not characteristic of HR, different resulting sequences can be produced by this pathway, making it a variable and generally unreliable chromosomal caretaker pathway when implemented alone. Accumulation of DNA repair errors in a NHEJ-only repair system due to mutations in either *BRCA1* or *BRCA2* increases likelihood of tumorigenesis in specific tissues. Additionally, deficiencies in functional *BRCA1* can lead to even less accurate repair by NHEJ based on the presence of larger, more significant deletions [72]. BRCA1’s involvement in both HR and NHEJ systems of DNA repair provides insight into how these pathways maintain genomic integrity, and more importantly, how deficiencies in key proteins change the ability of these pathways to promote cell survival, especially in cases of mutagenesis. Notably, in cells with *BRCA1* mutations, an impaired HR mechanism that results in dependence on an also dysfunctional NHEJ mechanism of repair is more likely to expedite tumorigenesis. The reliance of malignant cells on limited pathways of survival provides new, promising targets for treatment on the basis of synthetic lethality, demonstrated by the FDA-approved PARP inhibitor Olaparib. 

## 4. Repair-Independent BRCA1/2 Involvement

In addition to their critical roles in DNA repair, BRCA1 and BRCA2 are also implicated in conserving chromosomal integrity through their interactions in DNA replication, roles independent of homology-directed repair (HDR) [73]. Taglialetela et al. found that *BRCA1/2*-deficient cells showed significantly increased incidence of MRE11-mediated nascent DNA degradation and are therefore required for functional replication [74]. As in HR, BRCA2 chaperones RAD51 to DNA nascent strands to catalyze effective filling of gaps and allows the continuation of replication. This is demonstrated in an accumulation of RAD51 molecules at the site of collapsed replication forks [75]. Gaps in the nascent strands, caused by replication fork stalling secondary to replication stress, lead to a disruption in the replication process. Potentially, this disruption could precipitate from the uncoupling of the associated polymerase from the DNA helicase [76,77,78] or other perturbations, e.g., *BRCA1/2* deficiencies. BRCA2, as in HR, physically associates with RAD51, leading several RAD51 molecules to the stalled forks to initiate gap filling. This frees the replication fork and reestablishes replication; alternatively, fork regression may occur, resulting in formation of Holliday junctions [74,76]. Further, BRCA2-RAD51 catalyzes strand exchange interactions of single-strand DNA overhangs and gaps [76]. However, if stalled forks or gaps in DNA accumulate, forks will undergo reversal, forming a four-armed structure, which activates MRE11-dependent nascent DNA degradation via numerous fork remodeler proteins [73,74]. This MRN-independent involvement of BRCA1/2 is specific to replication, not repair, though the function of BRCA1/2 is similar between the two. Therefore, *BRCA1/2* mutations have the potential to affect both processes, which is an important consideration when designing effective therapies for *BRCA1/2* malignancies. The interdependence of DNA repair systems and DNA replication pathways on BRCA proteins could provide further therapeutic opportunity; however, more detailed studies addressing this topic are needed.

Defects in DNA replication, as in DNA repair mechanisms, are prominent in cells that are *BRCA1/2* deficient. Consequences of this deficiency include several pathological features extending beyond the accumulation of stalled replication forks, which include localization of cytosolic DNA forming micronuclei and abnormally progressing mitosis [79,80]. *BRCA1* deficiencies were linked to both chromosomal abnormalities following mitosis, most likely acquired following DNA replication, as well as increased incidence of stalled replication forks [81]. In cases of *BRCA2* deficiencies specifically, replication fork progression is significantly reduced with an accumulation of stalled forks, as described by Reisländer et al. [79,82,83]. The consequence of this pathological circumstance is the activation of MRE11-lead nucleolytic degradation in attempt to mitigate the stress of replication deterioration [79,84,85]. Additionally, these events prevent cell cycle progression into G2/M, which could provide cells the opportunity to implement compensatory mechanisms of replication fork restart or repair [79]. Appropriate implementation of alternative *BRCA*-independent pathways of fork restart/repair during impeded DNA replication could allow deficient cells to enter mitosis, maintain viability, and avoid entrance into apoptosis. Over time, however, *BRCA* deficiencies precipitate detectable levels of cytosolic nucleic acid accumulation in the form of micronuclei. These bodies activate recognition complexes in cells, as their accumulation raises cellular alarms upon perceived detection of “foreign” DNA. The aforementioned process results in activation of an immunogenic response [86], which involves upregulation of innate immunity agents, namely interferon-related elements, which initiate a cascade of complex immune-related events. Significant *BRCA1/2* involvement in critical cell processes independent of the DNA damage response renders cells particularly sensitive to inherited *BRCA* deficiencies, culminating in pathological fallout affecting several facets of cellular processes; furthermore, this reinforces the notion that many chromosomal caretaker pathways are reliant on *BRCA1/2*.

## 5. Traditional Therapeutic Options

*BRCA1/2* mutations play a significant role in determining clinical prognosis and survival curves in breast cancer patients. Zhu and colleagues published a meta-analysis in 2016 that indicates either *BRCA* mutation confers lower overall survival than in non-*BRCA*-mutated breast cancer [87]. In a hospital-based cohort, an absolute 10 years overall survival difference was demonstrated in *BRCA1/2* mutation carriers compared to noncarriers [88]. Further evidence supports increased hazard ratio with increased tumor grade, tumor size, and number of positive lymph nodes showing signs of lymphatic spread, though this is the case for both *BRCA-* and non-*BRCA*-mutated breast cancers [88]. Overall, there is sufficient evidence to conclude breast cancer in patients with *BRCA1/2* mutations carries a significantly worse prognosis.

Despite the unique challenges posed by *BRCA1/2* breast cancers, substantial progress in the development of effective therapies for several cancers has been made in recent decades. For breast cancer specifically, the traditional paradigm of treatment plans consisting of solely immediate interventional procedures and global cytotoxic chemotherapies has shifted to mechanistic models, including tissue-specific and tumor subtype-specific methods of targeted treatment adjunct to surgeries and radiation therapy [89]. In the case of BRCA1/2 breast tumors, either radical mastectomy or breast-conserving surgery (BCS) coupled with radiotherapy is a common starting point in the oncologic treatment plan following diagnosis [90]. Questions have been raised regarding which surgical procedure is more beneficial with respect to both long-term survival and quality of life for patients; interestingly, while BCS with radiotherapy is less invasive and traumatic than radical mastectomy, a 2010 study found there to be no significant difference in survival between these two surgical methods in patients with *BRCA1/2* breast cancers [91]. Additionally, there was no significant difference in outcomes of various operational interventions between breast cancer patients with and without *BRCA1/2* mutations [91]. While invasive surgical resection and DNA-targeting chemotherapeutics remain practical mainstays of treatment, especially for cancers that are particularly difficult to target or are more aggressive in nature, the goal of cancer therapeutic development is moving towards alternative, less traumatic means of tumor shrinkage and ultimately disease eradication. 

Chemotherapy targets cell replication machinery and DNA physical structure and often affects tissue systemically, causing a global effect. Platinum agents are cytotoxic chemotherapeutics that target rapidly dividing cells. Though cancerous cells inherently exhibit this trait, rapid division is not exclusive to malignant tissue—examples of these physiologic cells include those in hair follicles and intestinal epithelia. Two of the most widely used platinum agents implemented in breast cancer treatment include carboplatin and cisplatin, the former with larger leaving groups that correspond to a slightly lower rate of kinetic action and comparatively fewer side effects [92]. The mechanism of platinum agents involves the platinum metal compound interacting with purine bases in DNA of rapidly dividing cells, catalyzing DNA intrastrand crosslinking events, which significantly reduces the flexibility of DNA and causes replication fork interruptions in the course of DNA replication. This, in turn, leads to an accumulation of DNA damage to an extent that normal DNA repair machinery fails to mitigate. The severe degree of genomic damage activates a signaling cascade that ultimately results in induction of apoptosis [93]. Therefore, cells with compromised or at least significantly deficient DNA repair capacity, such as in *BRCA1/2* breast cancers, show increased sensitivity to these agents. Taxanes are another category of chemotherapy that function to inhibit mitosis in rapidly dividing cells by stabilizing tubulin monomers, effectively preventing microtubule disassembly, abrogating microtubule dynamics [90]. In contrast to platinum agents, microtubule-inhibiting agents such as taxanes are significantly less effective in *BRCA1/2*-mutated cancers, though the precise mechanism of resistance has not yet been identified [94]. In the case of platinum crosslinking agents and taxanes, an additional cytotoxic effect is observed in tumor tissue: Immunogenic Cell Death (ICD). This is accomplished through the activation of adaptive immune-related elements in response to certain anticancer therapies [95,96]. Platinum agents, specifically Cisplatin, display upregulation in the expression of Major Histocompatibility Complex I (MHC I) molecules and those linked to tumor antigen processing. Platinum agents also increase tumor sensitivity to programmed cell death protein 1 (PD-1) checkpoint inhibitors, seen in triple-negative tumors [96,97]. Taxanes also possess this ability to induce immune antitumor response, mainly through the elevated exposure of calreticulin on tumor cells; however, taxanes are less effective in stimulating ICD compared to platins [96,98]. Though solo chemotherapy generally lacks the ability to successfully and comprehensively target tumor cells, combining chemotherapy with other therapeutic options (e.g., surgical resection and radiation) generally results in a more pronounced disease response, while activating immune-mediated cytotoxic pathways and increasing tumor sensitization to other antitumor agents. 

Hormone-targeted therapies and the development of more advanced inhibitors show efficacy in treating breast cancers positive for estrogen (ER) and progesterone receptors (PR), as well as those that display the human epithelial growth factor-2 (HER2/neu) amplification. However, these treatment options are not commonly effective in the treatment of *BRCA1/2* breast cancers, which are typically “triple negative” with regard to presence of these targetable receptors [89]. The disparity between the rate of development of effective therapies for other breast cancers and for those predisposed by *BRCA1/2* mutation can likely be attributed to inability of physically inhibiting relevant target proteins, as is possible in other breast cancers. Although not mainstream, immunotherapies, especially Immune Checkpoint Blockade (ICB), are under evaluation for potential therapeutic application in the treatment of breast cancers. 

## 6. Immune Checkpoint Blockade Therapy

In recent years, ICB has emerged as a promising treatment strategy for various cancer types including breast cancer. ICB works by blocking immune checkpoints. Immune checkpoint molecules act as gatekeepers of immune responses. Cancer cells adopt these immune checkpoints to shut down immune responses and protect themselves, while application of ICB can reactivate immune responses against cancer. Cytotoxic T-lymphocyte–associated antigen 4 (CTLA-4), PD-1, and programmed cell death protein ligand 1 (PD-L1) are major targets for ICB development. Specifically, activation of CTLA-4 initiates binding to B7-1 and B7-2 on the surfaces of antigen presenting cells more tightly than CD28 receptors on the surface of T cells, which blunts T cell activation and effectively dampens the immune response to tumor antigens [99,100]. PD-1, a surface protein displayed on several immune cell types binds its ligand, PD-L1 or PD-L2 expressed on the membrane of cancer cells, also resulting in T cell deactivation [99,101,102]. Targeting inhibitory pathways such as these could address immune evasion of malignancies in various cancers. Since the first FDA approval of CTLA-4-blocking ipilimumab for melanoma in 2011, another 6 immune checkpoint inhibitors have been approved to treat more than a dozen different types of cancer [103,104,105]. In 2019, the FDA approved PDL-1 inhibitor, atezolizumab, in combination with paclitaxel for the treatment of metastatic triple-negative breast cancer in people whose tumors express PD-L1 based on data from a successful clinical trial [106]. Currently, many different checkpoint inhibitors are under development, both as monotherapy and in combination with other treatments, in a variety of cancer types. In spite of the unprecedented clinical success of ICB, optimizing and maximizing efficacy of ICB regimens remains a challenge.

## 7. Synthetic Lethality and Therapeutic Options

**Figure 4:**
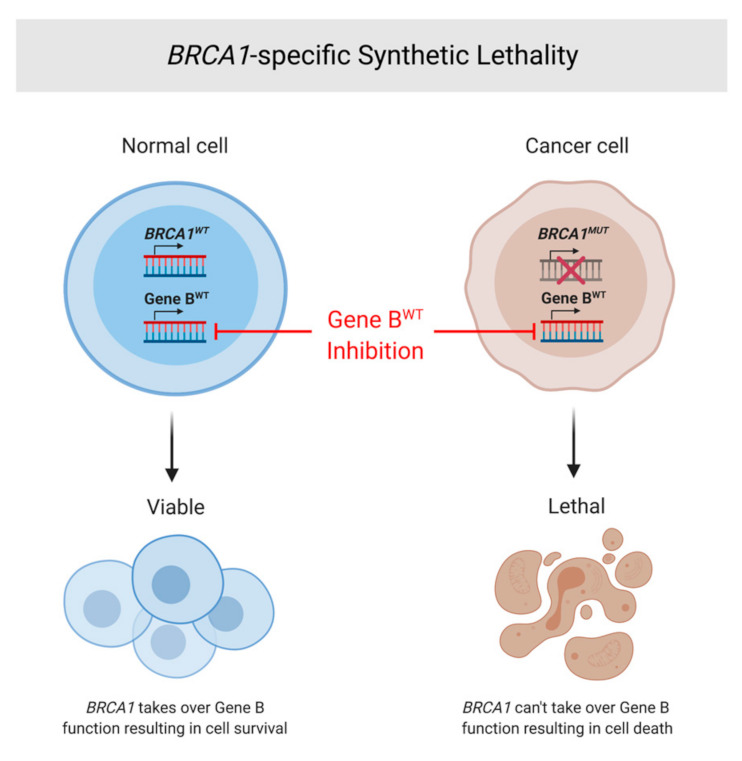
*BRCA1*-specific synthetic lethality. General explanation of synthetic lethality as applied to *BRCA1*. In the normal cell (**left**) with both WT *BRCA1* and WT Gene *B* (a SL partner of *BRCA1*), inhibition of Gene *B* will not affect cell viability. However, in a cancerous cell (**right**) with an oncogenic *BRCA1* perturbation and WT Gene *B*, inhibition of Gene *B* will lead to apoptotic cell death due to decreased functional capacity of the mutated BRCA1 protein.

Synthetic lethality denotes the relationship between two genes, or survival pathways, in a cell system whereby the silencing or deficiency of either gene retains cell viability, while the silencing of both genes results in cell death [20]. Elucidation of cell survival mechanisms and implicated genes is crucial to discovering a synthetic lethal partner of a gene of interest. In the context of *BRCA1-* and *BRCA2*-derived cancers, other genes involved in high-volume DNA repair, or those key in other critical pathways whose functionality is required for cell survival and proliferation, are potential contenders for *BRCA1/2* synthetic lethal partners (Figure 4). 

One confirmed *BRCA1* synthetic lethal partner, PARP, is an enzyme critical for the recognition and repair of single-strand DNA breaks (SSBs), and some DSBs, through base-excision repair (BER) [107]. While primarily associated with initiation of BER, PARP also plays a role in activating the NHEJ pathway. When PARP is inhibited, phosphorylation of DNA-pkcs is induced, which is the primary activation step in the NHEJ pathway [108]. Additionally, PARP has been implicated in other critical cell survival mechanisms including apoptosis regulation and epigenetic chromatin decondensation [109]; this multifactorial involvement in critical cell functions underscores the legitimacy of PARP as an integral regulatory gene, as well as it being a true synthetic lethal partner of *BRCA1* and potentially other genes involved in carcinogenesis. In BER, PARP binds to the site of the SSB, recruits several scaffold proteins including XRCC1, and auto poly ADP-ribosylates (autoPARylation) the break to signal additional repair proteins to repair the break [110,111]. ADP-ribosylation by PARP involves the addition of NAD+ to several targets of DNA repair, including scaffolding proteins recruited to the site of damage, PARP itself, and other nuclear targets [110]. This cascade of ADP-ribosylation by PARP is responsible for the signaling of DNA damage, propelling BER. 

**Figure 5:**
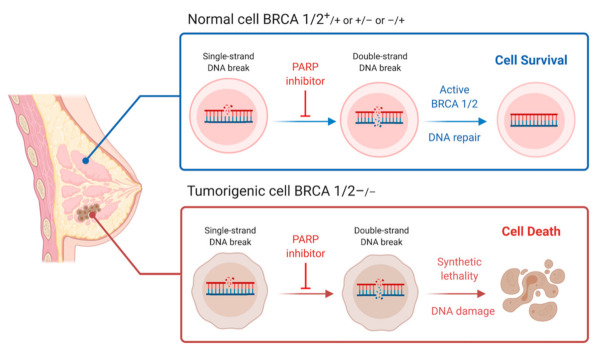
PARP inhibitors function on the basis of synthetic lethality. In the normal cell with physiologic *BRCA1/2* (above, blue), PARP inhibition will cause SSBs to progress to DSBs. However, functional *BRCA1/2* will have the capability to repair resulting DSB damage and maintain cell viability. In the tumorigenic cells (below, red), PARP inhibition will cause SSBs to progress to DSBs, but deficient BRCA1/2 protein levels will decrease rates of DSB repair and allow accumulation of DSBs—ultimately leading to cell death via apoptosis.

In 2018, the first drug therapy to function on the principle of synthetic lethality in *BRCA*-mutated metastatic breast cancer (and previously *BRCA*-mutated ovarian cancer) was approved by the FDA (Figure 5). Olaparib, the first FDA-approved clinically implemented PARP inhibitor (PARPi), acts by inhibiting the NAD+ binding pocket at the active sites of PARP1 and PARP2 [112]. This action “traps” PARP at the SSB and prevents autoPARylation, effectively terminating its signaling activity in DNA repair. Further studies suggest elevated efficacy of PARPi in combination with platinum therapy [113]. Synergistic effects of this drug combination induce *BRCA*-mutated cell sensitivity by exacerbating already deficient DNA repair mechanisms (effect of PARPi) while exploiting this deficiency by causing further destruction of DNA structure (effect of platinum therapy), ultimately accelerating tumor cell death. Newfound roles and relationships between other DNA repair mechanisms and PARP have been discovered since the release of the PARPis, which could explain the susceptibility of *BRCA1/2* breast cancer malignancies to PARPi therapy. Further, these discoveries introduce the potential of utilizing the principle of synthetic lethality, as well as PARPis, to other types of cancers [114]. Studies to expand the application of the PARPi to prostate and pancreatic cancers are currently underway [110].

Though PARP inhibition has led to significant success in the treatment of *BCRA1/2* breast cancer, more than 40% of *BRCA1/2*-deficient breast cancer patients are unresponsive to PARPi therapy [115]. Moreover, those who initially are responsive to therapy acquire PARPi resistance after prolonged use, leading to disease progression [16]. The elucidated mechanisms of PARPi resistance include PARPi efflux, restorative mutations recuing HR functions, PARP-freeing by upregulation of NAD+ synthesis, silencing of BP53, the introduction of point mutations in PARP, and increasing rate of replication after restabilization of the replication fork [116,117,118]. Though the exact mechanisms are unknown, one study found that loss of BP53 in cells leads to restoration of DSB repair through HR, mirroring the findings of recovered HR function in *BRCA*-mutated tumors, and conferring a degree of tumor resistance to PARPis [119]. These findings suggest that a reversion of the original *BRCA* mutation has taken place, either directly or indirectly, as a result of PARPi treatment, ultimately allowing reestablishment of a previously compromised survival pathway. There is also the possibility of additional, or secondary, mutations to the mutated *BRCA* gene that may partially restore HR functionality [120]. Uncovering the mechanisms of PARPi resistance is becoming increasingly necessary as resistance in *BRCA*-mutated cancers, and other cancers, emerges. Furthermore, ongoing research to elucidate novel synthetic lethal partners of *BRCA1/2* may allow for the development of therapies with greater efficacy and less potential for resistance. Additionally, researchers observed encouraging efficacy with the combination therapy of PARPi and cell cycle checkpoint inhibitors in breast and ovarian cancer patients. The cell cycle checkpoint proteins ataxia-telangiectasia-mutated- and Rad3-related kinase (ATR) and, its major downstream effector, checkpoint kinase 1 (CHK1) are activated in various cancer cells with damaged or incompletely replicated DNA. The activated ATR/CHK1 signaling causes cell cycle arrest and allows time for DNA repair [121,122]. ATR/CHK1 inhibitors prevent DNA damage induced cell cycle arrest, enable cancer cells with chromosome aberrations entry into mitosis, and eventually induce mitotic catastrophe and cell death. Preclinical studies have shown that cancer cells with defective DNA repair systems sensitize them to ATR/CHK1 inhibitors, which makes them ideal therapeutic targets. ATR/CHK1 inhibitors are currently developed either as monotherapy or in combination with chemotherapy or radiotherapy in preclinical and clinical studies [123,124,125,126,127]. It was reported that earlier generations of CHK1 inhibitors synergize with PARPi to kill breast cancer cells [128,129,130,131]. In a phase I clinical trial, one particular ATRi, VX-970, showed efficacy in patients with *BRCA1*-mutated ovarian cancer that was platinum refractory and PARPi resistant [127]. More recently, Dr. Simpkins’ group found that combination therapy of PARPi with ATR/CHK1 inhibitors showed a synergistic effect in *BRCA*-mutant ovarian cancer [132,133]. Interestingly, in preclinical studies with platinum-resistant ovarian cancer cell lines and PDX animal models, ATR or CHK1 inhibition can sensitize *BRCA*-deficient and PARPi-resistant cells [133]. Taken together, the above data highlight the importance of cell cycle checkpoint regulation of the cellular response to DNA replication stress. Like PARP, ATR/CHK1 is considered to be synthetically lethal with defective DNA repair [133,134]. The application of ATR/CHK1 inhibition to PARPi therapy in *BRCA*-deficient patients could potentiate increased DNA replication stress and provide further synthetic lethality synergism.

## 8. BRCA1/2 Ovarian Cancer

Ovarian cancer is the most lethal gynecologic malignancy and the second most common occurring overall [135,136,137]. Epithelial ovarian cancers are associated with a moderately strong genetic susceptibility, with heterozygous carriers of *BRCA1* and *BRCA2* germline mutations having increased lifetime risk of developing ovarian cancer of 40–60% and 11–30%, respectively [137,138,139]. Though ovarian malignancies are often diagnosed late in disease progression and typically have a poor prognosis, the utilization of *BRCA1* and *BRCA2* therapeutic targeting via PARPis bodes promising for future patient outcomes. 

Though *BRCA1* and *BRCA2* are most commonly associated with ovarian cancer as CPGs, several other CPGs have gained attention in recent years. Other tumor suppressor genes, including *CHEK2*, *TP53* (involved in Li–Fraumeni Syndrome), DSB repair genes, such as *RAD51*, and mismatch repair genes *MSH2* and *MLH1* (involved in Lynch Syndrome) have been strongly implicated in genetic predisposition to the development of ovarian cancer [140,141,142]. The heterogeneous nature of ovarian cancer makes classification and association of various CPGs with different forms of ovarian cancer daunting. Therefore, we focus on *BRCA1/2*-related mechanisms of carcinogenesis for high-grade serous epithelial cancer (HGSC), as high-grade ovarian carcinomas are most likely to demonstrate the greatest benefit from PARPi therapeutics among ovarian cancers. To support this notion, it is important to note that carcinomas comprise 90% of ovarian cancers, and that HR deficiency is estimated to be present in 50% of HGSCs [143]. 

The etiology of HGSC combines loss of intact dsDNA repair mechanisms with accumulation of a wide variety of genetic abnormalities, resulting in intratumoral heterogeneity [144]. This impairment of dsDNA break repair is similar to that which occurs in breast cancer pathogenesis in patients with *BRCA1/2* mutations. In vitro studies have demonstrated a functional interaction between *RAD51* and *BRCA1* whose combined DNA repair via HR plays an integral role in maintaining genomic stability; in the case of HGSC, maintenance of this stability is crucial in fallopian tube epithelium [145,146]. HGSC is a histological classification; however, it can be found in fallopian tube, ovarian, or peritoneal settings. More recently, support for serous tubal intraepithelial carcinoma (STIC), a generally noninvasive lesion of the distal fallopian tube, serving as a precursor lesion to HGSC has gained favor [147]. Dysplastic lesions of the fallopian tube have been demonstrated to occur more frequently in *BRCA1* carriers [147,148]. Though the consensus on what proportion of HGSC arises from fallopian versus ovarian primary lesions is still undetermined, it is clear that dysfunction in *BRCA1/2* and *RAD51* CPGs plays an important role in predisposing some patients to such pathologies. 

Metastasis of HSGC is unique in that it does not rely on blood or lymphatic dissemination, but instead can directly invade adjacent structures. Seeding of the peritoneal cavity is characteristic, and this only furthers poor prognosis of ovarian cancer whose presentation is typically late and insidious [149,150]. In vitro studies have implicated a role for PARP-1 induction of vascular endothelial growth factor (VEGF) angiogenesis in spread of epithelial ovarian cancer cells [151]. This supports pursuit of PARPi therapy further, as molecular targeting to prevent metastasis of this aggressive carcinoma would be novel and highly beneficial clinically. 

Most recently, combined strategy utilizing both a targeted approach (such as PARPi therapy) and immunotherapy (for example, monoclonal antibodies or checkpoint inhibitors such as PD-1 blockade) have been explored [152]. While PARPi therapy has been successfully employed as monotherapy for *BRCA1*-deficient ovarian tumors, there is less evidence supporting such a response in *BRCA1*-deficient breast cancer. Zhao and colleagues have uncovered a stimulator of interferon genes (STING)-dependent antitumor immune response (inducible by a STING agonist) that can reprogram the M2-like tumor associated macrophages (TAMs) predominant in breast tumor cells to the M1-like TAMs characteristic of ovarian tumors [152,153]. The study also highlights enhancement of Olaparib efficacy when combined with PD-1 blockade [153]. Altogether, we expect to see an increase in combining targeted and immunotherapy to further the effectiveness and breadth of PARPi use for treating susceptible tumors, primarily ovarian and breast cancers, but also potentially prostate and pancreatic tumors as well.

## 9. PARP Inhibitor Clinical Trails and Use in Practice

Since 2005, in vitro and in vivo studies have demonstrated that PARPis utilize the principle of synthetic lethality to prevent cell growth in the context of DNA repair defects [21,154,155]. Clinical studies have started to confirm the impressive impact of this therapeutic targeting; Olaparib has demonstrated success as both a primary and maintenance therapy for treatment of recurrent platinum sensitive ovarian cancer, while *Niraparib* and *Rucaparib* have also been FDA approved for second line maintenance [156,157]. Combining PARPis with ionizing radiation therapy has been shown to delay tumor growth in pre-clinical studies performed at Wayne State University for multiple cancer cell lines [158]. The sensitivity of tumors with *BRCA1/2* disruption demonstrated in clinical use of PARPis suggests similar success could be achieved with such a strategy for ovarian and breast cancers.

As of May 2021, 54 clinical trials were retrieved on ClinicalTrials.gov with search criteria of “PARPi”. Of these, 33 studies (whose statuses include not yet recruiting, recruiting, active, and completed trials) examine the efficacy of novel PARPi therapeutic use, while 3 studies examine treatment of PARPi-resistant cancers (the remaining 18 trials were excluded from this tabulation due to termination/withdrawal of study or lack of a measurement of PARPi efficacy as a primary outcome). Those examining efficacy of PARPi therapy include patients with ovarian, breast, endometrial, uterine leiomyosarcoma, fallopian, primary peritoneal, pancreatic, prostate, head and neck, and nasopharyngeal carcinomas (Table 2). Currently, only one of these 33 trials has published results, but the number of studies in progress is encouraging for patients with susceptible tumors, especially those with HR deficiency.

In addition to these ongoing clinical trials, two Cochrane reviews are currently available regarding PARP inhibitors, with the first reviewing PARPi therapy for epithelial ovarian cancer published in 2016, and the second reviewing support for PARPi use in treatment breast cancer, just recently published in April 2021. Four randomized controlled trials involving women with epithelial ovarian cancer are synthesized to reveal improvement of progression-free survival with addition of Olaparib to traditional therapy, though no significant improvement in overall survival is observed [160]. The authors conclude PARP inhibitors should play a role in treating patients with ovarian carcinoma. Such a definitive conclusion is not drawn by the authors who review the role of PARPi use for breast cancer. A small progression-free survival advantage is similarly found for patients with locally advanced/metastatic HER2-negative, *BRCA* germline-mutated breast cancer; however, the authors are more hesitant to implore strong recommendations for clinical use, particularly as monotherapy, perhaps due to the emergence of other meta-analyses that investigate different PARP inhibitors [161]. The authors conclude: “From the current information available, including the addition of our systematic review results, it remains difficult to know which PARP inhibitor to choose for which patient” [161]. Ultimately, both reviews address a shortage of data due to the small number of trials, excluding the possibility for meta-analysis at the time of each publication. However, expansion of sample size and increasing the number of trials will surely allow for more powerful clinical recommendations to be made in the coming years; just in the last year, meta-analyses of randomized control trials demonstrated an association between PARPis and progression-free survival in patients with high-grade epithelial ovarian cancer [162,163]. These controlled trials overall support the clinical utilization of PARP inhibitors as an element of therapeutic regimen for both ovarian and breast carcinoma to enhance progression-free survival; however, monotherapy appears to be supported for appropriate ovarian cancers only at this time.

## 10. Conclusions

Across various fields within medicine, the combination of genomic sequencing advances with rapidly expanding knowledge about cellular signaling pathways has welcomed an age of targeted therapy, and trends in cancer therapeutics exemplify this shift. The hereditary nature of detrimental cancers only reinforces the necessity of developing targeted formulations that intervene in CPG mutations. While we describe the structure, mechanisms, and relevance of several CPGs in this review, including *PALB2*, *BARD1*, *TP53*, *BRIP1*, and *RAD51*, the role of *BRCA1/2*, particularly *BRCA1*, in directing DSB repair towards the HR pathway deserves special attention when considering which CPG targets warrant prioritization. In addition, the prevalent nature of breast cancer occurrence is an important factor to consider, as an estimated 12.9% of women born today will develop breast cancer some time in their lives [164]. The current approach of targeted therapeutics for breast cancer is multifaceted, ranging from targeted interventional radiology procedures that utilize percutaneous ablation to hormone-based chemotherapy, such as Trastuzumab (Herceptin) that treats HER2 receptor-positive cancers [165]. However, breast cancer patients with *BRCA1/2* mutations tend to acquire triple-negative tumors that are not susceptible to newly popular hormone receptor targeting. Because *BRCA1/2* mutations are the most common CPGs altered in breast cancer, yet such mutations have thus far proven to be directly non-druggable, there is a significant need for the development of alternative therapeutic approach for these cancers. 

We believe synthetic lethality has the potential to drive discovery of novel cancer therapies, especially for those that stem from genetic mutations that are not directly targetable. The reliance of cell survival on alternative DNA repair mechanisms when key repair genes, such as *BRCA1/2*, are mutated, likely plays a significant role in persistence of many tumor types, including breast and ovarian. Targeting these alternative pathways has demonstrated efficacy in inhibiting tumor growth through in vitro, in vivo, and clinical studies. There is justified excitement about PARPis, one of the first successful applications of synthetic lethality to cancer treatment in patients. However, we have already become aware of some limitations of these drugs. 

PARPis have demonstrated substantial efficacy in *BRCA1/2* breast and ovarian cancers until the point of tumor resistance, after which point progression of disease ensues, and the once effective therapy is essentially useless in affected patients. Additionally, because PARPis have demonstrated several modalities of resistance, including drug efflux, reversion mutations to *BRCA1*, PARP-freeing, and replication fork re-stabilization, *BRCA1* tumors have several possible avenues to escape attack by this particular therapy. This indicates a need for additional studies to better elicit resistance mechanisms and address these obstacles either in the form of a new generation of PARPis, or by discovering new therapeutic targets with less potential for this type of resistance. 

Other challenges that may arise with the development of more PARPis and other very specifically targeted therapies are ethical and decision making-related questions. With the three PARPis currently on the market for treatment or maintenance therapy for ovarian cancer, there are few disqualifying criteria among patients, yet there is acknowledgement within the field that the efficacy of therapies is often only studied for certain types of tumors, e.g., HGSC [166]. Therefore, unless clear, explicit decision-making guidelines are written, clinicians will be left guessing whether such a therapy would have similar efficacy in a patient with clear-cell, low-grade, or mucinous ovarian carcinoma. Additionally, though having more choices of precision therapies may be advantageous for the breadth of cancers that can be addressed, individual physician discretion when selecting between two targeted drugs that treat the same type of tumor is less ideal than establishing clear criteria based on randomized control clinical data. However, such trials are time-consuming and may be challenging due to the heterogeneous nature of different tumor types and indications for unique therapies, as exemplified in ovarian cancer.

Due to the limitations conferred by tumor resistance to novel drugs and the considerations enumerated above, we strongly advocate for further work discovering synthetic lethal partners of *BRCA1/2* as well as other non-druggable CPGs. There are various approaches to identifying synthetic lethal partners in the literature; the advent of CRISPR has expanded opportunities in gene target exploration and has advanced the field greatly [167]. When trying to discover new synthetic lethal partners, a broad to narrow approach is favorable; that is, considering different pathways that may interact in currently unelucidated mechanisms may lead to further knowledge and novel discoveries. One particular approach of interest would be utilizing the Mining Synthetic Lethals algorithm, which is a software programmed to mine pan-cancer human primary tumor data to identify SL partners for specific cancers [168]. The database includes tumor data for thousands of mutations and has shown success in predicting synthetic lethal partners [169]. This demonstrates the utility of cross-disciplinary work, specifically bioinformatics, in the hunt for synthetic lethal partners that have potential to direct future targeted cancer therapy. Ultimately, we hope to see future discovery of synthetic lethal partners for non-druggable yet incredibly high-risk CPGs, such as *BRCA1/2*, and to witness such discoveries’ impacts on targeted cancer therapeutics and prevention in a way that is comprehensive of different tumor types.

## Figures and Tables

**Table 1:** Breast cancer predisposition genes confer varying risk for carcinogenesis due to their role in critical molecular processes.

Breast Cancer Predisposition Genes (CPGs)			
Risk Level	Gene	Estimated Incidence of Mutations in Hereditary Breast Cancer	Associated Process
**High Risk**	*BRCA1*	~50% between *BRCA1* and *BRCA2* [4]	HR (several functions) [6,8]
	*BRCA2*	~50% between *BRCA1* and *BRCA2* [4]	HR (loading and leading RAD51) [6,8]
	*PALB2*	<3% [9,10]	HR (recruitment of BRCA2 and DNA replication via ICL) [6]
	*BARD1*	<1% [8,11]	HR (ubiquitin ligase) [6]
**Moderate Risk**	*TP53*	25–30% [12,13]	Cell cycle checkpoint protein (triggered by DNA damage) [7]
	*BRIP1*	<3% [9,10]	HR (complexes with BRCA1) [7]
	*RAD51C*	<3% [9,10]	HR (promotes invading strand exchange) [8]
	*MSH6*	<1% [8]	Mismatch repair (MMR)-associated protein [14]
**Low Risk**	*ATM*	<3% [9,10]	Cell cycle checkpoint protein [7]
	*CHEK2*	<3% [9,10]	Cell cycle checkpoint protein (checkpoint kinase-2) [7]
	*Nbn*	<1% [8]	DDR (complexes with MRE11 and RAD50 in DSB) [15]
	*RAD50*	<1% [8]	DDR (complexes with MRE11 and Nbn in DSB) [15]

**Table 2:** Clinical trials investigating the efficacy of PARP inhibitors in various solid malignancies are increasing, with a majority currently in progress.

Trial Name	Status	Conditions	Phase	Results Summary
Efficacy and Safety of PARPi to Treat Pancreatic Cancer	Unknown	Pancreatic Cancer	Phase II	None available yet
Survival Data and Characteristics of Finisterian Patients Treated with PARP Inhibitors for Ovarian Cancer Between 2014 and 2019	Completed	Ovarian Neoplasm	Does not specify	None available
Pamiparib in Fusion Positive, Reversion Negative HGSOC or Carcinosarcoma With BRCA1/2 Gene Mutations If Progression on PARPI or Chemotherapy	Recruiting	Ovarian Cancer Carcinosarcoma	Phase II	None available yet
A Study to Examine Olaparib Maintenance Retreatment in Patients with Epithelial Ovarian Cancer	Active, not recruiting	Epithelial Ovarian Cancer	Phase III	None available yet
Study of M4344 in Combination with Niraparib	Not yet recruiting	Advanced Solid Tumor Breast Cancer	Phase I Phase II	None available yet
Window of Opportunity Trial, PARP Inhibitor Rucaparib Affect on PD-L1 Expression in Triple Negative Breast Tumors	Recruiting	Breast Cancer	Early Phase I	None available yet
Olaparib Arsenic Trioxide Platinum Resistance Relapsed Ovarian Cancer	Active, not recruiting	Ovarian Cancer	Phase I Phase II	None available yet
Recurrent Ovarian Carcinosarcoma Anti-pd-1 Niraparib	Recruiting	Ovarian Carcinosarcoma Endometrial Carcinosarcoma	Phase II Phase III	None available yet
ATR Inhibitor BAY 1895344 Plus Niraparib Phase 1b Study in Advanced Solid Tumors and Ovarian Cancer	Recruiting	Advanced Solid Tumors (excluding prostate cancer) Ovarian Cancer	Phase I	None available yet
The Clinical Markers for PARP Inhibitors-related Efficacy in Ovarian Cancer	Recruiting	Ovarian Cancer	Does not specify	None available yet
Platinum and PARPI for Neoadjuvant Treatment of Triple Negative Breast Cancer (TNBC) and/or Germline BRCA (gBRCA) Positive Breast Cancer	Recruiting	Breast Cancer	Phase II Phase III	None available yet
Induction and Maintenance Treatment with PARP Inhibitor and Immunotherapy in HPV-negative HNSCC	Recruiting	Head and Neck Squamous Cell Carcinoma	Phase II	None available yet
Stratified Evaluation of PDS and NACT-IDS in Ovarian Cancer (FOCUS)	Not yet recruiting	Epithelial Ovarian Cancer Fallopian Tube Cancer Primary Peritoneal Carcinoma	Phase III	None available yet
Niraparib Maintenance in Patients with Advanced Ovarian Cancer at Neoadjuvant Setting	Recruiting	Ovarian Cancer	Phase II	None available yet
Combination of HX008 and Niraparib in Germ-line-mutated Metastatic Breast Cancer	Not yet recruiting	Breast Cancer	Phase II	None available yet
Pembrolizumab and Olaparib in Recurrent/Metastatic, Platinum Resistant Nasopharyngeal Cancer	Not yet recruiting	Nasopharyngeal Carcinoma	Phase II	None available yet
Anlotinib and Niraparib Dual Therapy Evaluation in Platinum-resistant Recurrent Ovarian Cancer	Recruiting	Platinum-Resistant Ovarian Cancer	Phase II	None available yet
Multi-maintenance Olaparib After Disease Recurrence in Participants with Platinum Sensitive *BRCA* HGSOC	Active, not recruiting	Ovarian Cancer	Early Phase I	None available yet
Olaparib After Response to Trabectedin-pegylated Liposomal Doxorubicin in Recurrent Ovarian Carcinoma	Active, not recruiting	Ovarian Cancer	Phase II	None available yet
Investigation of 2X-121 in Patients with Advanced Ovarian Cancer Selected by the 2X-121 DRP	Recruiting	Advanced Ovarian Cancer	Phase II	None available yet
DVAC/OvCa and Standard of Care (SoC) in Relapsed Ovarian, Fallopian Tube, and Primary Peritoneal Carcinoma	Not yet recruiting	Ovarian Cancer Fallopian Tube Cancer Peritoneal Cancer	Phase III	None available yet
A Study of Niraparib in Patients with Ovarian Cancer Who Have Received Three or Four Previous Chemotherapy Regimens	Active, not recruiting	Ovarian Neoplasms Ovarian Cancer	Phase II	Of 463 participants who received Niraparib, 122 completed study. Of 47 patients in primary efficacy population, 13 (28%) achieved overall response [159]
Olaparib and Temozolomide in Treating Patients with Advanced, Metastatic, or Unresectable Uterine Leiomyosarcoma	Active, not recruiting	Stage III–IV Uterine Corpus Leiomyosarcoma	Phase II	None available yet
Analysis of the Clinical Experience with Rucaparib in the Rucaparib Access Program (RAP) in Spain—A GEICO Study	Recruiting	Epithelial Ovarian Cancer Fallopian Tube Cancer Primary Peritoneal Cancer	Does not specify	None available yet
A Study to Evaluate Rucaparib in Patients with Solid Tumors and With Deleterious Mutations in HRR Genes	Recruiting	Solid Tumor	Phase II	None available yet
A Study in Ovarian Cancer Patients Evaluating Rucaparib and Nivolumab as Maintenance Treatment Following Response to Front-Line Platinum-Based Chemotherapy	Active, not recruiting	Epithelial Ovarian Cancer Primary Peritoneal Fallopian Tube Cancer	Phase III	None available yet
A Study of Rucaparib Versus Physician’s Choice of Therapy in Patients with Metastatic Castration-resistant Prostate Cancer and Homologous Recombination Gene Deficiency	Recruiting	Metastatic Castration-Resistant Prostate Cancer	Phase III	None available yet
A Study of Rucaparib in Patients with Metastatic Castration-resistant Prostate Cancer and Homologous Recombination Gene Deficiency	Active, not recruiting	Metastatic Castration-Resistant Prostate Cancer	Phase II	None available yet
A Study of ZEN003694 and Talazoparib in Patients With Triple Negative Breast Cancer	Recruiting	Triple -Negative Breast Cancer	Phase II	None available yet
An Efficacy and Safety Study of Niraparib in Men With Metastatic Castration-Resistant Prostate Cancer and DNA-Repair Anomalies	Active, not recruiting	Prostatic neoplasms	Phase II	None available yet
Ascending Doses of Ceralasertib in Combination With Chemotherapy and/or Novel Anti Cancer Agents	Recruiting	Advanced Solid Malignancies—Head and Neck Squamous Cell Carcinoma, ATM Pro/Deficient Non-Small-Cell Lung Cancer, Gastric, Breast, and Ovarian Cancer	Phase I Phase II	None available yet
Olaparib With or Without Atezolizumab in Treating Patients With Locally Advanced Unresectable or Metastatic Non-HER2-Positive Breast Cancer	Recruiting	Locally Advanced Unresectable Breast Carcinoma Metastatic Breast Carcinoma Stage III–IV Breast Cancer	Phase II	None available yet
Niraparib and Dostarlimab for the Treatment of Germline or Somatic BRCA1/2 and PALB2 Mutated Metastatic Pancreatic Cancer	Recruiting	Metastatic Pancreatic Ductal Adenocarcinoma Stage IV Pancreatic Cancer	Phase II	None available yet

## Data Availability

Not applicable.

## References

[1] Bray F., Ferlay J., Soerjomataram I., Siegel R.L., Torre L.A., Jemal A. (2018). Global cancer statistics 2018: GLOBOCAN estimates of incidence and mortality worldwide for 36 cancers in 185 countries. CA Cancer J. Clin..

[2] Dossus L., Benusiglio P.R. (2015). Lobular breast cancer: Incidence and genetic and non-genetic risk factors. Breast Cancer Res..

[3] Bayraktar S., Glück S. (2012). Systemic therapy options in *BRCA* mutation-associated breast cancer. Breast Cancer Res. Treat..

[4] Kuchenbaecker K.B., Hopper J.L., Barnes D.R., Phillips K.A., Mooij T.M., Roos-Blom M.J., Jervis S., Van Leeuwen F.E., Milne R.L., Andrieu N. (2017). Risks of breast, ovarian, and contralateral breast cancer for *BRCA1* and *BRCA2* mutation carriers. JAMA J. Am. Med. Assoc..

[5] Antoniou A.C., Casadei S., Heikkinen T., Barrowdale D., Pylkäs K., Roberts J., Lee A., Subramanian D., De Leeneer K., Fostira F. (2014). Breast-Cancer Risk in Families with Mutations in *PALB2*. N. Engl. J. Med..

[6] Easton D.F. (1999). How many more breast cancer predisposition genes are there?. Breast Cancer Res..

[7] Sato K., Koyasu M., Nomura S. (2017). Mutation status of *RAD51C*, *PALB2* and *BRIP1* in 100 Japanese familial breast cancer cases without *BRCA1* and *BRCA2* mutations. Cancer Sci..

[8] Shimelis H., LaDuca H., Hu C. (2018). Triple-negative breast cancer risk genes identified by multigene hereditary cancer panel testing. J. Natl. Cancer Inst..

[9] Filippini S.E., Vega A. (2013). Breast cancer genes: Beyond *BRCA1* and *BRCA2*. Front. Biosci..

[10] Pharoah P.D.P., Antoniou A.C., Easton D.F., Ponder B.A.J. (2008). Polygenes, Risk Prediction, and Targeted Prevention of Breast Cancer. N. Engl. J. Med..

[11] Śniadecki M., Brzeziński M., Darecka K. (2020). Bard1 and breast cancer: The possibility of creating screening tests and new preventive and therapeutic pathways for predisposed women. Genes.

[12] Silwal-Pandit L., Langerød A., Børresen-Dale A.L. (2017). *TP53* mutations in breast and ovarian cancer. Cold Spring Harb. Perspect Med..

[13] Børresen-Dale A.L. (2003). *TP53* and breast cancer. Hum. Mutat..

[14] Roberts M.E., Jackson S.A., Susswein L.R. (2018). *MSH6* and *PMS2* germ-line pathogenic variants implicated in Lynch syndrome are associated with breast cancer. Genet. Med..

[15] Kim J.H., Penson A.V., Taylor B.S., Petrini J.H.J. (2019). Nbn−Mre11 interaction is required for tumor suppression and genomic integrity. Proc. Natl. Acad. Sci. USA.

[16] Murthy P., Muggia F. (2019). Women’s cancers: How the discovery of *BRCA* genes is driving current concepts of cancer biology and therapeutics. Ecancermedicalscience.

[17] Liu E.Y., Xu N., O’Prey J. (2015). Loss of autophagy causes a synthetic lethal deficiency in DNA repair. Proc. Natl. Acad. Sci. USA.

[18] Sancar A., Lindsey-Boltz L.A., Ünsal-Kaçmaz K., Linn S. (2004). Molecular mechanisms of mammalian DNA repair and the DNA damage checkpoints. Annu. Rev. Biochem..

[19] Epstein R.J. (2013). The unpluggable in pursuit of the undruggable: Tackling the dark matter of the cancer therapeutics universe. Front. Oncol..

[20] O’Neil N.J., Bailey M.L., Hieter P. (2017). Synthetic lethality and cancer. Nat. Rev. Genet..

[21] Farmer H., McCabe H., Lord C.J. (2005). Targeting the DNA repair defect in *BRCA* mutant cells as a therapeutic strategy. Nature.

[22] Tutt A., Robson M., Garber J.E. (2010). Oral poly(ADP-ribose) polymerase inhibitor olaparib in patients with *BRCA1* or *BRCA2* mutations and advanced breast cancer: A proof-of-concept trial. Lancet.

[23] Deng C.X., Brodie S.G. (2000). Roles of BRCA1 and its interacting proteins. BioEssays.

[24] Bielinska B., Bielinska B.A. (2017). The *BRCA1* tumor suppressor: Potential long-range interactions of the *BRCA1* promoter and the risk of breast cancer. Rev. Artic J. Transl. Sci. J. Transl. Sci..

[25] Semmler L., Reiter-Brennan C., Klein A. (2019). *BRCA1* and breast cancer: A review of the underlying mechanisms resulting in the tissue-specific tumorigenesis in mutation carriers. J. Breast Cancer.

[26] Prakash R., Zhang Y., Feng W., Jasin M. (2015). Homologous recombination and human health: The roles of BRCA1, BRCA2, and associated proteins. Cold Spring Harb. Perspect Biol..

[27] Christou C.M., Kyriacou K. (2013). BRCA1 and its network of interacting partners. Biology.

[28] Gorodetska I., Kozeretska I., Dubrovska A. (2019). *BRCA* genes: The role in genome stability, cancer stemness and therapy resistance. J. Cancer.

[29] Clark S.L., Rodriguez A.M., Snyder R.R., Hankins G.D.V., Boehning D. (2012). Structure-function of the tumor suppressor *BRCA1*. Comput. Struct. Biotechnol. J..

[30] Godet I., MGilkes D. (2017). *BRCA1* and *BRCA2* mutations and treatment strategies for breast cancer. Integr. Cancer Sci. Ther..

[31] Sy S.M.H., Huen M.S.Y., Chen J. (2009). PALB2 is an integral component of the BRCA complex required for homologous recombination repair. Proc. Natl. Acad. Sci. USA.

[32] Zhang F., Ma J., Wu J. (2009). PALB2 Links BRCA1 and BRCA2 in the DNA-Damage Response. Curr. Biol..

[33] Wu S., Zhou J., Zhang K. (2020). Molecular Mechanisms of PALB2 Function and Its Role in Breast Cancer Management. Front. Oncol..

[34] Miki Y., Swensen J., Shattuck-Eidens D. (1994). A strong candidate for the breast and ovarian cancer susceptibility gene *BRCA1*. Science.

[35] Wu L.C., Wang Z.W., Tsan J.T. (1996). Identification of a RING protein that can interact in vivo with the *BRCA1* gene product. Nat. Genet..

[36] Adamovich A.I., Banerjee T., Wingo M. (2019). Functional analysis of *BARD1* missense variants in homology-directed repair and damage sensitivity. PLoS Genet..

[37] Ayi T.C., Tsan J.T., Hwang L.Y., Bowcock A.M., Baer R. (1998). Conservation of function and primary structure in the BRCA1-associated RING domain (BARD1) protein. Oncogene.

[38] Venkitaraman A.R. (2002). Cancer susceptibility and the functions of *BRCA1* and *BRCA2*. Cell.

[39] Kolinjivadi A.M., Sannino V., de Antoni A., Técher H., Baldi G., Costanzo V. (2017). Moonlighting at replication forks – a new life for homologous recombination proteins BRCA1, BRCA2 and RAD51. FEBS Lett..

[40] Powell S.N., Kachnic L.A. (2003). Roles of *BRCA1* and *BRCA2* in homologous recombination, DNA replication fidelity and the cellular response to ionizing radiation. Oncogene.

[41] Tischkowitz M., Xia B. (2010). PALB2/FANCN: Recombining cancer and fanconi anemia. Cancer Res..

[42] Nepomuceno T.C., De Gregoriis G., de Oliveira F.M.B., Suarez-Kurtz G., Monteiro A.N., Carvalho M.A. (2017). The role of *PALB2* in the DNA damage response and cancer predisposition. Int. J. Mol. Sci..

[43] Zhang F., Fan Q., Ren K., Andreassen P.R. (2009). PALB2 functionally connects the breast cancer susceptibility proteins BRCA1 and BRCA2. Mol. Cancer Res..

[44] Xia B., Sheng Q., Nakanishi K. (2006). Control of BRCA2 Cellular and Clinical Functions by a Nuclear Partner, PALB2. Mol. Cell..

[45] Chen Q., Van Der Sluis P.C., Boulware D., Hazlehurst L.A., Dalton W.S. (2005). The FA/BRCA pathway is involved in melphalan-induced DNA interstrand cross-link repair and accounts for melphalan resistance in multiple myeloma cells. Blood.

[46] Cimmino F., Formicola D., Capasso M. (2017). Dualistic role of *BARD1* in cancer. Genes.

[47] Irminger-Finger I., Jefford C.E. (2006). Is there more to *BARD1* than *BRCA1*?. Nat. Rev. Cancer.

[48] Daza-Martin M., Densham R.M., Morris J.R. (2019). BRCA1-BARD1: The importance of being in shape. Mol. Cell Oncol..

[49] Densham R.M., Garvin A.J., Stone H.R. (2016). Human BRCA1-BARD1 ubiquitin ligase activity counteracts chromatin barriers to DNA resection. Nat. Struct Mol. Biol..

[50] Bunting S.F., Callén E., Wong N. (2010). 53BP1 inhibits homologous recombination in *BRCA1*-deficient cells by blocking resection of DNA breaks. Cell.

[51] Levran O., Attwooll C., Henry R.T. (2005). The BRCA1-interacting helicase BRIP1 is deficient in Fanconi anemia. Nat. Genet..

[52] Yu X., Chini C.C.S., He M., Mer G., Chen J. (2003). The BRCT Domain Is a Phospho-Protein Binding Domain. Science.

[53] Cantor S.B., Bell D.W., Ganesan S. (2001). BACH1, a novel helicase-like protein, interacts directly with BRCA1 and contributes to its DNA repair function. Cell.

[54] Cantor S., Drapkin R., Zhang F. (2004). The BRCA1-associated protein BACH1 is a DNA helicase targeted by clinically relevant inactivating mutations. Proc. Natl. Acad. Sci. USA.

[55] Barnes J.L., Zubair M., John K., Poirier M.C., Martin F.L. (2018). Carcinogens and DNA damage. Biochem. Soc. Trans..

[56] Jackson S.P. (2002). Sensing and repairing DNA double-strand breaks. Carcinogenesis.

[57] Scully R., Panday A., Elango R., Willis N.A. (2019). DNA double-strand break repair-pathway choice in somatic mammalian cells. Nat. Rev. Mol. Cell Biol..

[58] Lundin C., Erixon K., Arnaudeau C. (2002). Different Roles for Nonhomologous End Joining and Homologous Recombination following Replication Arrest in Mammalian Cells. Mol. Cell Biol..

[59] Wright W.D., Shah S.S., Heyer W.D. (2018). Homologous recombination and the repair of DNA double-strand breaks. J. Biol. Chem..

[60] Ingram S.P., Warmenhoven J.W., Henthorn N.T. (2019). Mechanistic modelling supports entwined rather than exclusively competitive DNA double-strand break repair pathway. Sci. Rep..

[61] Onyango D.O., Lee G., Stark J.M. (2017). PRPF8 is important for BRCA1-mediated homologous recombination. Oncotarget.

[62] Daley J.M., Sung P. (2014). 53BP1, BRCA1, and the Choice between Recombination and End Joining at DNA Double-Strand Breaks. Mol. Cell Biol..

[63] Ceccaldi R., Rondinelli B., D’Andrea A.D. (2016). Repair Pathway Choices and Consequences at the Double-Strand Break. Trends Cell Biol..

[64] Liu Y., Lu L.Y. (2020). BRCA1 and homologous recombination: Implications from mouse embryonic development. Cell Biosci..

[65] Lieber M.R. (2010). The mechanism of double-strand DNA break repair by the nonhomologous DNA end-joining pathway. Annu. Rev. Biochem..

[66] Burma S., Chen B.P.C., Chen D.J. (2006). Role of non-homologous end joining (NHEJ) in maintaining genomic integrity. DNA Repair.

[67] Smith G.C.M., Jackson S.P. (1999). The DNA-dependent protein kinase. Genes Dev..

[68] Doherty A.J., Jackson S.P. (2001). DNA repair: How Ku makes ends meet. Curr. Biol..

[69] Walker J.R., Corpina R.A., Goldberg J. (2001). Structure of the Ku heterodimer bound to dna and its implications for double-strand break repair. Nature.

[70] Martin I.V., MacNeill S.A. (2002). ATP-dependent DNA ligases. Genome Biol..

[71] Calsou P., Delteil C., Frit P., Drouet J., Salles B. (2003). Coordinated assembly of Ku and p460 subunits of the DNA-dependent protein kinase on DNA ends is necessary for XRCC4-ligase IV recruitment. J. Mol. Biol..

[72] Zhuang J., Zhang J., Willers H. (2006). Checkpoint kinase 2-mediated phosphorylation of BRCA1 regulates the fidelity of nonhomologous end-joining. Cancer Res..

[73] Kolinjivadi A.M., Sannino V., De Antoni A. (2017). Smarcal1-Mediated Fork Reversal Triggers Mre11-Dependent Degradation of Nascent DNA in the Absence of Brca2 and Stable Rad51 Nucleofilaments. Mol. Cell.

[74] Taglialatela A., Alvarez S., Leuzzi G. (2018). HHS Public Access. Restoration.

[75] Daboussi F., Courbet S., Benhamou S. (2008). A homologous recombination defect affects replication-fork progression in mammalian cells. J. Cell Sci..

[76] Costanzo V. (2011). Brca2, Rad51 and Mre11: Performing balancing acts on replication forks. DNA Repair.

[77] Lehmann A.R., Fuchs R.P. (2006). Gaps and forks in DNA replication: Rediscovering old models. DNA Repair.

[78] Lopes M., Foiani M., Sogo J.M. (2006). Multiple mechanisms control chromosome integrity after replication fork uncoupling and restart at irreparable UV lesions. Mol. Cell..

[79] Reisländer T., Lombardi E.P., Groelly F.J. (2019). BRCA2 abrogation triggers innate immune responses potentiated by treatment with PARP inhibitors. Nat. Commun..

[80] Lemaçon D., Jackson J., Quinet A. (2017). MRE11 and EXO1 nucleases degrade reversed forks and elicit MUS81-dependent fork rescue in *BRCA2*-deficient cells. Nat. Commun..

[81] Tarsounas M., Sung P. (2020). The antitumorigenic roles of BRCA1–BARD1 in DNA repair and replication. Nat. Rev. Mol. Cell Biol..

[82] Zimmer J., Tacconi E.M.C., Folio C. (2016). Targeting *BRCA1* and *BRCA2* Deficiencies with G-Quadruplex-Interacting Compounds. Mol. Cell..

[83] Lai X., Broderick R., Bergoglio V. (2017). MUS81 nuclease activity is essential for replication stress tolerance and chromosome segregation in BRCA2-deficient cells. Nat. Commun..

[84] Michl J., Zimmer J., Buffa F.M., McDermott U., Tarsounas M. (2016). *FANCD2* limits replication stress and genome instability in cells lacking *BRCA2*. Nat. Struct Mol. Biol..

[85] Schlacher K., Christ N., Siaud N., Egashira A., Wu H., Jasin M. (2011). Double-strand break repair-independent role for BRCA2 in blocking stalled replication fork degradation by MRE11. Cell.

[86] Vanpouille-Box C., Demaria S., Formenti S.C., Galluzzi L. (2018). Cytosolic DNA Sensing in Organismal Tumor Control. Cancer Cell.

[87] Zhu Y., Wu J., Zhang C. (2016). *BRCA* mutations and survival in breast cancer: An updated systematic review and meta-analysis. Oncotarget.

[88] Schmidt M.K., Van Den Broek A.J., Tollenaar R.A.E.M. (2017). Breast Cancer Survival of *BRCA1/BRCA2* Mutation Carriers in a Hospital-Based Cohort of Young Women. J. Natl. Cancer Inst..

[89] Harbeck N., Gnant M. (2017). Breast cancer. Lancet.

[90] Lee A., Moon B.I., Kim T.H. (2020). *BRCA1/BRCA2* pathogenic variant breast cancer: Treatment and prevention strategies. Ann. Lab. Med..

[91] Pierce L.J., Phillips K.A., Griffith K.A. (2010). Local therapy in *BRCA1* and *BRCA2* mutation carriers with operable breast cancer: Comparison of breast conservation and mastectomy. Breast Cancer Res. Treat..

[92] Makovec T. (2019). Cisplatin and beyond: Molecular mechanisms of action and drug resistance development in cancer chemotherapy. Radiol. Oncol..

[93] Ghosh S. (2019). Cisplatin: The first metal based anticancer drug. Bioorg. Chem..

[94] Sung M., Giannakakou P. (2014). *BRCA1* regulates microtubule dynamics and taxane-induced apoptotic cell signaling. Oncogene.

[95] Galluzzi L., Vitale I., Aaronson S.A. (2018). Molecular mechanisms of cell death: Recommendations of the Nomenclature Committee on Cell Death 2018. Cell Death Differ..

[96] Fumet J.D., Limagne E., Thibaudin M., Ghiringhelli F. (2020). Immunogenic cell death and elimination of immunosuppressive cells: A double-edged sword of chemotherapy. Cancers.

[97] Voorwerk L., Slagter M., Horlings H.M. (2019). Immune induction strategies in metastatic triple-negative breast cancer to enhance the sensitivity to PD-1 blockade: The TONIC trial. Nat. Med..

[98] Hodge J.W., Garnett C.T., Farsaci B. (2013). Chemotherapy-induced immunogenic modulation of tumor cells enhances killing by cytotoxic T lymphocytes and is distinct from immunogenic cell death. Int. J. Cancer.

[99] Tan T.J., Chan J.J., Kamis S., Dent R.A. (2018). What is the role of immunotherapy in breast cancer?. Chin. Clin. Oncol..

[100] Wolchok J.D., Saenger Y. (2008). The Mechanism of Anti-CTLA-4 Activity and the Negative Regulation of T-Cell Activation. Oncologist.

[101] Keir M.E., Butte M.J., Freeman G.J., Sharpe A.H. (2008). PD-1 and its ligands in tolerance and immunity. Annu. Rev. Immunol..

[102] Chen D.S., Mellman I. (2017). Elements of cancer immunity and the cancer-immune set point. Nature.

[103] Robert C. (2020). A decade of immune-checkpoint inhibitors in cancer therapy. Nat. Commun..

[104] Hargadon K.M., Johnson C.E., Williams C.J. (2018). Immune checkpoint blockade therapy for cancer: An overview of FDA-approved immune checkpoint inhibitors. Int. Immunopharmacol..

[105] Vaddepally R.K., Kharel P., Pandey R., Garje R., Chandra A.B. (2020). Review of indications of FDA-approved immune checkpoint inhibitors per NCCN guidelines with the level of evidence. Cancers.

[106] Schmid P., Adams S., Rugo H.S. (2018). Atezolizumab and Nab-Paclitaxel in Advanced Triple-Negative Breast Cancer. N. Engl. J. Med..

[107] Turk A.A., Wisinski K.B. (2018). PARP inhibitors in breast cancer: Bringing synthetic lethality to the bedside. Cancer.

[108] Patel A.G., Sarkaria J.N., Kaufmann S.H. (2011). Nonhomologous end joining drives poly(ADP-ribose) polymerase (PARP) inhibitor lethality in homologous recombination-deficient cells. Proc. Natl. Acad. Sci. USA.

[109] Kraus M., Alimzhanov M.B., Rajewsky N., Rajewsky K. (2004). Survival of resting mature B lymphocytes depends on BCR signaling via the Igα/β heterodimer. Cell.

[110] Lee J.M., Ledermann J.A., Kohn E.C. (2014). PARP Inhibitors for *BRCA1/2* mutation-associated and *BRCA*-like malignancies. Ann. Oncol..

[111] Morales J.C., Li L., Fattah F.J. (2014). Review of poly (ADP-ribose) polymerase (PARP) mechanisms of action and rationale for targeting in cancer and other diseases. Crit. Rev. Eukaryot. Gene Expr..

[112] Bochum S., Berger S., Martens U.M. (2018). Olaparib. Recent Results Cancer Res..

[113] Lord C.J., Ashworth A. (2017). PARP inhibitors: Synthetic lethality in the clinic. Science.

[114] Pascal J.M. (2018). The comings and goings of PARP-1 in response to DNA damage. DNA Repair.

[115] Bartek J., Lukas J. (2003). Chk1 and Chk2 kinases in checkpoint control and cancer. Cancer Cell.

[116] Thomas A., Murai J., Pommier Y. (2018). The evolving landscape of predictive biomarkers of response to PARP inhibitors. J. Clin. Investig..

[117] D’Andrea A.D. (2018). Mechanisms of PARP inhibitor sensitivity and resistance. DNA Repair.

[118] Pettitt S.J., Krastev D.B., Brandsma I. (2018). Genome-wide and high-density CRISPR-Cas9 screens identify point mutations in PARP1 causing PARP inhibitor resistance. Nat. Commun..

[119] Jaspers J.E., Kersbergen A., Boon U. (2013). Loss of 53BP1 causes PARP inhibitor resistance in *BRCA1*-mutated mouse mammary tumors. Cancer Discov..

[120] Sakai W., Swisher E.M., Karlan B.Y. (2008). Secondary mutations as a mechanism of cisplatin resistance in *BRCA2*-mutated cancers. Nature.

[121] Booth L., Cruickshanks N., Ridder T., Dai Y., Grant S., Dent P. (2013). PARP and CHK inhibitors interact to cause DNA damage and cell death in mammary carcinoma cells. Cancer Biol. Ther..

[122] Booth L., Roberts J., Poklepovic A., Dent P. (2018). The CHK1 inhibitor SRA737 synergizes with PARP1 inhibitors to kill carcinoma cells. Cancer Biol. Ther..

[123] Chen C.C., Kennedy R.D., Sidi S., Look A.T., D’Andrea A. (2009). CHK1 inhibition as a strategy for targeting fanconi anemia (FA) DNA repair pathway deficient tumors. Mol. Cancer.

[124] Huntoon C.J., Flatten K.S., Wahner Hendrickson A.E. (2013). ATR inhibition broadly sensitizes ovarian cancer cells to chemotherapy independent of *BRCA* status. Cancer Res..

[125] Hur J., Ghosh M., Kim T.H. (2021). Synergism of AZD6738, an ATR inhibitor, in combination with belotecan, a camptothecin analogue, in chemotherapy-resistant ovarian cancer. Int. J. Mol. Sci..

[126] Kim H., George E., Ragland R.L. (2017). Targeting the ATR/CHK1 axis with PARP inhibition results in tumor regression in *BRCA*-mutant ovarian cancer models. Clin. Cancer Res..

[127] Kim H., Xu H., George E. (2020). Combining PARP with ATR inhibition overcomes PARP inhibitor and platinum resistance in ovarian cancer models. Nat. Commun..

[128] Krajewska M., Fehrmann R.S.N., Schoonen P.M. (2015). ATR inhibition preferentially targets homologous recombination-deficient tumor cells. Oncogene.

[129] Li H., Liu Z.Y., Wu N., Chen Y.C., Cheng Q., Wang J. (2020). PARP inhibitor resistance: The underlying mechanisms and clinical implications. Mol. Cancer.

[130] Mei L., Zhang J., He K., Zhang J. (2019). Ataxia telangiectasia and Rad3-related inhibitors and cancer therapy: Where we stand. J. Hematol. Oncol..

[131] Mengwasser K.E., Adeyemi R.O., Leng Y. (2019). Genetic Screens Reveal *FEN1* and *APEX2* as *BRCA2* Synthetic Lethal Targets. Mol. Cell..

[132] Mitchell C., Park M., Eulitt P., Yang C., Yacoub A., Dent P. (2010). Poly(ADP-Ribose) polymerase 1 modulates the lethality of CHK1 inhibitors in carcinoma cells. Mol. Pharmacol..

[133] O’Carrigan B., de Miguel Luken M.J., Papadatos-Pastos D. (2016). Phase I trial of a first-in-class ATR inhibitor VX-970 as monotherapy (mono) or in combination (combo) with carboplatin (CP) incorporating pharmacodynamics (PD) studies. J. Clin. Oncol..

[134] Qiu Z., Oleinick N.L., Zhang J. (2018). ATR/CHK1 inhibitors and cancer therapy. Radiother. Oncol..

[135] Siegel R.L., Miller K.D., Jemal A. (2016). Cancer statistics, 2016. CA Cancer J. Clin..

[136] Pan Z., Xie X. (2017). *BRCA* mutations in the manifestation and treatment of ovarian cancer. Oncotarget.

[137] Moschetta M., George A., Kaye S.B., Banerjee S. (2016). *BRCA* somatic mutations and epigenetic *BRCA* modifications in serous ovarian cancer. Ann. Oncol..

[138] Easton D.F., Ford D., Bishop D.T. (1995). Breast and ovarian cancer incidence in *BRCA1*-mutation carriers. Am. J. Hum. Genet..

[139] Ford D., Easton D.F., Stratton M. (1998). Genetic Heterogeneity and Penetrance Analysis of the *BRCA1* and *BRCA2* Genes in Breast Cancer Families. Am. J. Hum. Genet..

[140] Toss A., Tomasello C., Razzaboni E. (2015). Hereditary ovarian cancer: Not only *BRCA1* and *2* Genes. Biomed Res. Int..

[141] Walsh T., Casadei S., Coats K.H. (2006). Spectrum of mutations in *BRCA1*, *BRCA2*, *CHEK2*, and *TP53* in families at high risk of breast cancer. J. Am. Med. Assoc..

[142] Nevanlinna H., Bartek J. (2006). The *CHEK2* gene and inherited breast cancer susceptibility. Oncogene.

[143] Dos Santos E.S., Lallemand F., Petitalot A., Caputo S.M., Rouleau E. (2020). Hrness in breast and ovarian cancers. Int. J. Mol. Sci..

[144] Singh N., McCluggage W.G., Gilks C.B. (2017). High-grade serous carcinoma of tubo-ovarian origin: Recent developments. Histopathology.

[145] George S.H.L., Shaw P. (2014). *BRCA* and early events in the development of serous ovarian cancer. Front. Oncol..

[146] Scully R., Chen J., Plug A. (1997). Association of BRCA1 with Rad51 in mitotic and meiotic cells. Cell.

[147] Kim J., Park E.Y., Kim O. (2018). Cell origins of high-grade serous ovarian cancer. Cancers.

[148] Piek J.M.J., Van Diest P.J., Zweemer R.P. (2001). Dysplastic changes in prophylactically removed Fallopian tubes of women predisposed to developing ovarian cancer. J. Pathol..

[149] Lisio M.A., Fu L., Goyeneche A., Gao Z.H., Telleria C. (2019). High-grade serous ovarian cancer: Basic sciences, clinical and therapeutic standpoints. Int. J. Mol. Sci..

[150] Bast R.C., Hennessy B., Mills G.B. (2009). The biology of ovarian cancer: New opportunities for translation. Nat. Rev. Cancer.

[151] Wei W., Li Y., Lv S., Zhang C., Tian Y. (2016). PARP-1 may be involved in angiogenesis in epithelial ovarian cancer. Oncol. Lett..

[152] Zhao J.J. (2021). Panel to Discuss How Combination Therapies Could Create Better, Longer-Lasting Outcomes. https://www.aacrnews.org/news/panel-to-discuss-how-combination-therapies-could-create-better-longer-lasting-outcomes/.

[153] Zhang X., Chiang H.C., Wang Y. (2017). Attenuation of RNA polymerase II pausing mitigates BRCA1-associated R-loop accumulation and tumorigenesis. Nat. Commun..

[154] Bryant H.E., Schultz N., Thomas H.D. (2005). Specific killing of *BRCA2*-deficient tumours with inhibitors of poly(ADP-ribose) polymerase. Nature.

[155] Pillay N., Tighe A., Nelson L. (2019). DNA Replication Vulnerabilities Render Ovarian Cancer Cells Sensitive to Poly(ADP-Ribose) Glycohydrolase Inhibitors. Cancer Cell.

[156] Moore K., Colombo N., Scambia G. (2018). Maintenance Olaparib in Patients with Newly Diagnosed Advanced Ovarian Cancer. N. Engl. J. Med..

[157] Arend R., Westin S.N., Coleman R. (2020). Decision analysis for secondline maintenance treatment of platinum sensitive recurrent ovarian cancer: A review. Int. J. Gynecol. Cancer.

[158] Jannetti S.A., Zeglis B.M., Zalutsky M.R., Reiner T. (2020). Poly(ADP-Ribose)Polymerase (PARP) Inhibitors and Radiation Therapy. Front. Pharmacol..

[159] Moore K.N., Secord A.A., Geller M.A. (2019). Niraparib monotherapy for late-line treatment of ovarian cancer (QUADRA): A multicentre, open-label, single-arm, phase 2 trial. Lancet Oncol..

[160] Walker L.C., Lattimore V.L., Kvist A. (2019). Comprehensive Assessment of BARD1 Messenger Ribonucleic Acid Splicing With Implications for Variant Classification. Front Genet..

[161] Taylor A.M., Chan D.L.H., Tio M. (2021). PARP (Poly ADP-Ribose Polymerase) inhibitors for locally advanced or metastatic breast cancer. Cochrane Database Syst. Rev..

[162] Mirza M.R., Coleman R.L., González-Martín A. (2020). The forefront of ovarian cancer therapy: Update on PARP inhibitors. Ann. Oncol..

[163] Lin Q., Liu W., Xu S. (2021). PARP inhibitors as maintenance therapy in newly diagnosed advanced ovarian cancer: A meta-analysis. BJOG Int. J. Obstet. Gynaecol..

[164] Cancer Statistics Review, 1975-2017-SEER Statistics. https://seer.cancer.gov/archive/csr/1975_2017/.

[165] Kenny L.M., Orsi F., Adam A. (2017). Interventional radiology in breast cancer. Breast.

[166] Clinical Challenges: PARP Inhibitors in Ovarian Cancer | MedPage Today. https://www.medpagetoday.com/clinical-challenges/asco-ovarian-cancer/87068.

[167] Wang T., Yu H., Hughes N.W. (2017). Gene Essentiality Profiling Reveals Gene Networks and Synthetic Lethal Interactions with Oncogenic Ras. Cell.

[168] Sinha S., Thomas D., Chan S. (2017). Systematic discovery of mutation-specific synthetic lethals by mining pan-cancer human primary tumor data. Nat. Commun..

[169] Xiao Y., Thakkar K.N., Zhao H. (2020). The m6A RNA demethylase FTO is a HIF-independent synthetic lethal partner with the *VHL* tumor suppressor. Proc. Natl. Acad. Sci. USA.

